# Coding of odors in the anterior olfactory nucleus

**DOI:** 10.14814/phy2.14284

**Published:** 2019-11-28

**Authors:** Takahiro Tsuji, Chiharu Tsuji, Maja Lozic, Mike Ludwig, Gareth Leng

**Affiliations:** ^1^ Centre for Discovery Brain Sciences University of Edinburgh Edinburgh UK; ^2^ Department of Immunology Centre for Neuroendocrinology University of Pretoria Pretoria South Africa

**Keywords:** anterior olfactory nucleus, hazard function, interspike interval, oscillatory firing, vasopressin

## Abstract

Odorant molecules stimulate olfactory receptor neurons, and axons of these neurons project into the main olfactory bulb where they synapse onto mitral and tufted cells. These project to the primary olfactory cortex including the anterior olfactory nucleus (AON), the piriform cortex, amygdala, and the entorhinal cortex. The properties of mitral cells have been investigated extensively, but how odor information is processed in subsequent brain regions is less well known. In the present study, we recorded the electrical activity of AON neurons in anesthetized rats. Most AON cells fired in bursts of 2–10 spikes separated by very short intervals (<20 ms), in a period linked to the respiratory rhythm. Simultaneous recordings from adjacent neurons revealed that the rhythms of adjacent cells, while locked to the same underlying rhythm, showed marked differences in phase. We studied the responses of AON cells to brief high‐frequency stimulation of the lateral olfactory tract, mimicking brief activation of mitral cells by odor. In different cells, such stimuli evoked transient or sustained bursts during stimulation or, more commonly, post‐stimulation bursts after inhibition during stimulation. This suggests that, in AON cells, phase shifts occur as a result of post‐inhibitory rebound firing, following inhibition by mitral cell input, and we discuss how this supports processing of odor information in the olfactory pathway. Cells were tested for their responsiveness to a social odor (the bedding of a strange male) among other simple and complex odors tested. In total, 11 cells responded strongly and repeatedly to bedding odor, and these responses were diverse, including excitation (transient or sustained), inhibition, and activation after odor presentation, indicating that AON neurons respond not only to the type of complex odor but also to temporal features of odor application.

## Abstract 2

The output cells of the main olfactory bulb project via the lateral olfactory tract (LOT) to the anterior olfactory nucleus (AON), but how AON neurons process that information is not well understood. We recorded the electrical activity of AON neurons in anaesthetized rats, and studied their responses to diverse odors and to electrical stimulation of the LOT. Most AON cells fired in bursts linked to the respiratory rhythm. The rhythms of adjacent cells, while locked to the same underlying rhythm, often showed marked differences in phase. Some AON cells showed short latency excitation following stimulation of the LOT, indicating that they were primary recipients of afferents from the olfactory bulb; these all fired burst during inspiration. However, most AON cells were inhibited by LOT stimulation, indicating that they received inhibitory projections from the primary recipients, and most of these cells fired bursts during expiration. We discuss the implications of these observations for the coding of odor information.

## Graphical abstract

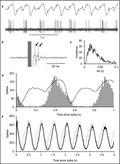

## 1 INTRODUCTION

Olfactory information is transduced when odorant molecules contact the dendrites of olfactory receptor neurons in the olfactory epithelium of the dorsal nasal cavity (Buck & Axel, 1991). These neurons project to the glomerular layer of the main olfactory bulb (MOB) (Mombaerts et al., 1996) where they synapse onto the dendrites of mitral and tufted cells, the output neurons of the olfactory bulb (Lledo, Gheusi, & Vincent, 2005; Menini, Lagostena, & Boccaccio, 2004; Shipley & Ennis, 1996). Each mitral cell sends its apical dendrite to a single glomerulus, and this receives inputs from a single class of olfactory receptor neurons (Bargmann, 2006); the mitral cells are thus tuned to respond to odorants that activate a single type of odorant receptor. However, the olfactory sensory neurons are also mechanosensitive (Grosmaitre, Santarelli, Tan, Luo, & Ma, 2007) and are activated rhythmically by respiratory activity (Duchamp‐Viret, Kostal, Chaput, Lansky, & Rospars, 2005). Accordingly, the spontaneous spiking activity of many mitral cells follows the respiratory cycle, with peak firing rates occurring typically late in the inspiratory phase in rats (Buonviso et al., 2003; Margrie & Schaefer, 2003). In the presence of an odor that activates (or inhibits) the mitral cell, this cyclic activity is amplified (or attenuated). Thus for each mitral cell, odor information is coded as the amplitude (relative to a basal amplitude) of a signal that oscillates with the respiratory rhythm.

By contrast to mitral cells, tufted cells are more excitable, and respond with a shorter latency to stimulation of olfactory sensory neurons (Burton & Urban, 2014; Geramita & Urban, 2017; Griff, Mafhouz, & Chaput, 2008; Vaaga & Westbrook, 2017), hence those that show a clear respiratory rhythm are active earlier in the respiratory cycle than mitral cells, and whereas mitral cells show sustained responses to sensory stimulation, the responses of tufted cells adapt.

Axons from the mitral and tufted cells converge to form the lateral olfactory tract (LOT), and collaterals of these axons innervate the anterior olfactory nucleus (AON) an important second‐order processing station in the olfactory pathway (Brunjes, Illig, & Meyer, 2005; Sosulski, Bloom, Cutforth, Axel, & Datta, 2011). Individual cells in the AON receive inputs from mitral cells whose dendrites arise in different glomeruli, and hence they are more broadly tuned than mitral or tufted cells. Transynaptic labeling studies in mice suggest that individual AON cells receive inputs from at least four mitral cells (Miyamichi et al., 2011). In mice, it appears that mitral and tufted cells may project to different regions of the AON (Igarashi et al., 2012).

In urethane‐anesthetized rats, mitral cells show complex patterns of spontaneous activity: the mean firing rate is about 6.4 spikes/s, and comprises long bursts (of ~154s) at ~11 spikes/s separated by long silent periods (~ 100s) that are not synchronized among even neighboring mitral cells (Leng, Hashimoto, Tsuji, Sabatier, & Ludwig, 2014). Within bursts, activity in most mitral cells oscillates with a period of 400–600 ms, reflecting peak activation during the inspiration phase of respiration, and excitatory responses to odors are typically apparent as an intensification of this respiratory‐locked oscillatory discharge. The significance of the long bursting appears to be that cells that alternate between activity and silence can optimally detect both excitatory stimuli (in the silent phase) and inhibitory stimuli (in the active phase).

The AON is a very heterogeneous nucleus. It contains pyramidal glutamatergic cells and a variety of interneurons, many of which are GABA‐ergic, but which also contain a considerable diversity of neuropeptides (Kay & Brunjes, 2014). One role of the AON is to assemble the inputs of activated glomeruli into a representation of complex odorants, and one particular role for the AON may be in processing socially relevant complex odors. Whereas the output cells of the MOB respond selectively to a narrow range of odors, in mice most pyramidal AON neurons can be activated by mixtures of structurally dissimilar components (Lei, Mooney, & Katz, 2006). The response of an AON neuron to an effective mixture often exceeds the sum of its responses to the components, indicating a nonlinear combinatorial interaction.

Here, we used in vivo electrophysiology to study how inputs from the olfactory bulb are processed in the AON. We studied the spontaneous discharge patterning of AON neurons to identify subpopulations and studied their responsiveness to stimulation of the LOT. To investigate neuronal responses to a social odor, we impregnated air with the odor from the soiled bedding of the cage of a novel adult male rat and applied this odor in an air stream to the nose as well as testing other simple and complex odors.

## 2 MATERIALS AND METHODS

### 2.1 Ethical approval

Procedures conducted in the UK were approved by the local Ethics Committee and the UK Home Office under the Animals Scientific Procedures Act 1986.

### 2.2 Electrophysiology

**Figure 1:**
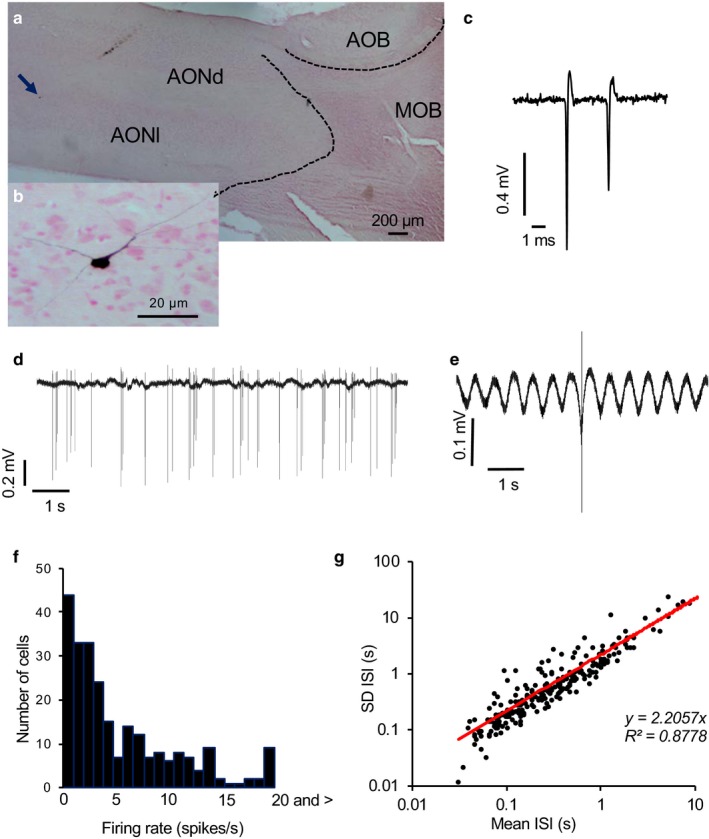
Juxtacellularly labeled neuron in the AON. (a) Juxtacellularly labeled neuron (arrowhead) in the rat AON shown at higher magnification on the lower. AOB; accessory olfactory bulb, MOB; main olfactory bulb, AONd; anterior olfactory nucleus dorsal, AONl; anterior olfactory nucleus lateral. (b) Higher magnification of the neuron arrowed in A showing multipolar neuron with oval cell body. (c) Extract of voltage trace showing individual spikes. This neuron showed decreasing spike height in spikes clustered at high frequency. (d) Voltage record spontaneous activity pattern showing rhythmic discharge of spikes, including short bursts. (e) Average action potential from this neuron (average of all spikes fired during 300 s) expanded to show rhythmic oscillations of voltage. (f) Distribution of spontaneous firing rates of a sample of 240 AON neurons. (g) The *SD* of ISIs is closely correlated with the mean ISI (*R* ^2^ = .88)

Single neurons were recorded from 120 adult male Sprague‐Dawley rats (250–400 g) anesthetized with urethane (ethyl carbamate, 1.3 g/kg i.p.) using conventional extracellular recording techniques. In some experiments, respiration was monitored using a Pulse Transducer (TN1012/ST, ADInstruments, Oxford, UK). A recording electrode (glass micropipette filled with 0.9% NaCl, 20–40 MΩ) was lowered into the dorsally exposed AON, 1.0–2.5 mm lateral to the midline and 3.8–4.7 mm anterior to bregma. We recorded neural activity through an amplifier (Axopatch 200b, Molecular Device, Sunnyvale, USA) and processed them by Spike2 software (Cambridge Electronic Design Limited, Cambridge, UK). In initial experiments, the position of the recording electrode was verified by visualizing incorporated neurobiotin in the recorded cell by juxtacellular labeling (Sabatier & Leng, 2008) (Figure 1a,b). Labeled cells were consistently found in the main, ventrolateral portion of the AON, and were multipolar with oval cell bodies.

To stimulate the LOT, a concentric bipolar stimulating electrode (SNEX‐100, Clark Electromedical Instrument, Kent, UK) was positioned on the ipsilateral LOT, via a burr hole in the dorsal surface of the skull, 1.4 mm posterior to bregma, 3.2 mm lateral to midline, 9.5 mm deep (Paxinos & Watson, 2006). Electrical stimulation (1 ms matched biphasic pulses, 1 mA peak‐to‐peak) was generated by a GRASS S88 stimulator with stimulus isolation and constant current units (Grass Products, Warwick, USA) as previously (Leng et al., 2014). In initial experiments, the position of the stimulating electrode was confirmed histologically and by verifying that stimulation at this site evoked the expected potential changes in the mitral cell layer of the main olfactory bulb (Leng et al., 2014).

In some cases, double recordings were made with the single recording electrode. When the spike heights were sufficiently different, these were sorted using the LabSpike software generated by Bhumbra, Inyushkin, and Dyball (2004) and available from the CED website (http://www.ced.co.uk/upu.shtml).

Odors were applied through a polythene cannula (0.1 mm diameter) placed 3 mm in front of the nose of the rat. Beddings were from male rat cages different from the cages of the experimental rats. For each test, 20 ml of odor‐saturated air was applied to the nose over ~5s using a 50 ml syringe. Some cells were tested with a range of odors, including heptanal, hexanal, valderaldehyde, lemon, garlic, and peppermint, and when responses were observed, the specificity was checked by a similar application of odor‐free air. For cells that showed a response to bedding odor, activity in 0.5 s bins was compared 20 s before and in the first 10 s after bedding was applied.

### 2.3 Statistical analysis

Autocorrelation histograms, ISI distributions, and peri‐stimulus time histograms were constructed in Spike2. Autocorrelation histograms were constructed from 300 s of spontaneous activity in 100 ms bins, and normalized autocorrelation histograms were normalized to the total number of spikes. ISI histograms were constructed in 10 ms bins from at least 300 s of stable spontaneous discharge activity. To generate population averages (consensus ISI distributions), each ISI distribution was normalized to the total number of ISIs. We measured index of dispersion (IoD) of firing rates in 10 s bins over 300 s of spontaneous activity for each cell as the ratio of the variance to the mean.

We used two ways to assess the relationship of spiking activity to respiratory rhythm. First, we looked at spike activity relative to the respiratory cycle, using the wavemark function in Spike2 to mark each respiratory cycle at a consistent stage, and using this as a trigger, constructed a histogram of spike activity relative to this trigger and used the same trigger to plot the average respiratory waveform. Second, we looked at the respiratory waveform relative to spike activity, using each recorded spike as a trigger for the waveform average: this spike‐triggered waveform is thus constructed in a way strictly analogous to the construction of the autocorrelation histogram.

## 3 RESULTS

Our first objective was to describe the spontaneous firing patterns of AON neurons and test them for responsiveness to bedding odor and for their responsiveness to LOT stimulation. We initially analyzed 240 spontaneously active AON cells recorded from 103 rats. Their firing rates ranged from 0.1 to 32.4 spikes/s, with a mean (*SEM*) of 5.8 ± 0.37 spikes/s. Although the spontaneous firing rates were thus generally low, instantaneous frequencies in most cells occasionally exceeded 100 spikes/s (Figure 1c) indicating that the low spontaneous rates did not reflect an inability to discharge at high frequencies. Most cells fired in intermittent short bursts (Figure 1d) that often were associated with rhythmic changes in the background field potential (Figure 1e). The firing rates of AON cells were distributed unimodally, and subpopulations could not be distinguished from this alone (Figure 1f). For AON cells generally, the standard deviation of ISIs was proportional to the mean firing rate, revealing no subpopulations (Figure 1g).

### 3.1 Rhythmic and arrhythmic cells

**Figure 2:**
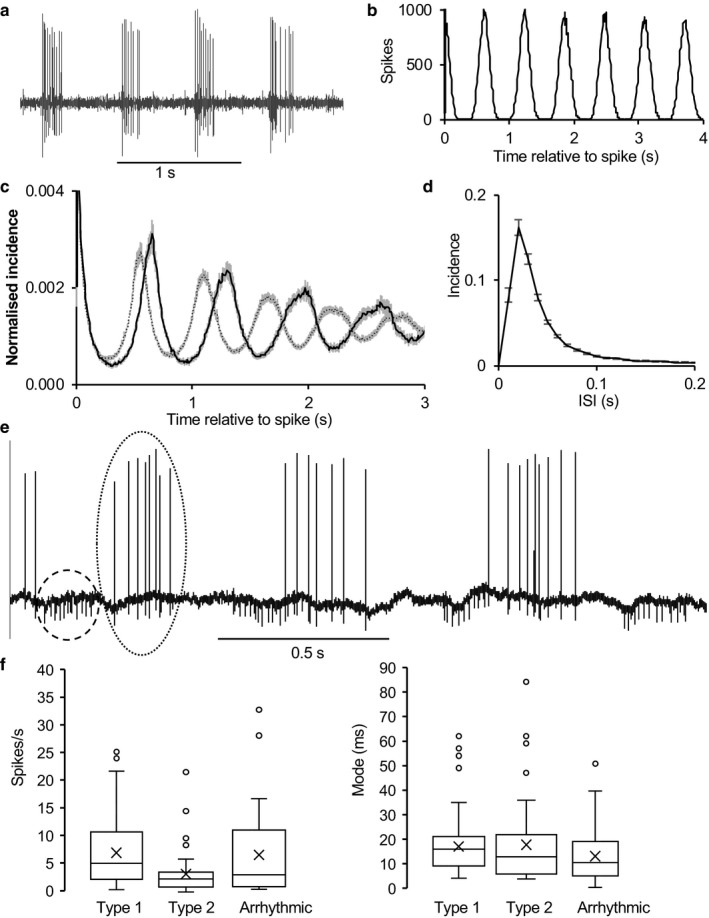
Type 1 neurons in the AON. (a) Extract of voltage trace of a typical type 1 neuron. (b) Autocorrelation of spike activity of the neuron in A showing discharge activity with a period of ~600 ms. (c) Normalized autocorrelations from type 1 neurons sorted according to period of rhythm (500–600 ms dotted line, *n* = 67; 600–700 ms solid line, *n* = 50). Means ± SE. (d) Consensus ISI distribution for 143 type 1 cells (means ± SE). (e) Pair of type 1 neurons, displaying bursts with a period of about 600 ms. Bursts in the smaller cell (one burst circled in dashed line) are out of phase with bursts in the larger cell (circled in dotted line). (f) Box plots showing mean and interquartile ranges, median (X) and outliers (circles) for spontaneous firing rates (left and modal ISI (right) for the three groups of AON neurons

For each cell, we constructed autocorrelation histograms over 300 s or more of stable spontaneous activity (Figure 2b). This allowed us to identify three large subpopulations. The largest of these, 147 *type 1 rhythmic cells* (Figure 2b), fired at 6.8 ± 0.5 spikes/s (range 0.18–25.1) and showed cyclic activity characterized by regular peaks in the autocorrelation histogram separated by intervals of a constant duration (mean 0.61 ± 0.008 s; Figure 2c). This is similar to the periodicity of rhythmic oscillations in mitral cells and corresponds to the mean respiratory rate of urethane‐anesthetized rats (85–110 min^−1^). Activity mainly comprised bursts of 2–10 spikes separated by ISIs of <25 ms in each phase of the cycle. About half (70) of the type 1 cells showed virtually no activity between bursts, while the remainder showed sparse spikes between bursts. The ISI distributions of type 1 cells were all similarly skewed and were unimodal in the range 0–200 ms with a mean mode of 17.1 ± 0.9 ms (median 16), (Figure 2d,f). Four cells discharged with periods of 300–400 ms, approximately half that of the mean respiratory rhythm (not shown). In some recordings, spikes from two neurons could be readily separated by spike height and waveform. In all cases (10 pairs), the two cells were locked to the same underlying rhythm, but their discharge activity could be either in phase or out of phase with each other (Figure 2e).

**Figure 3:**
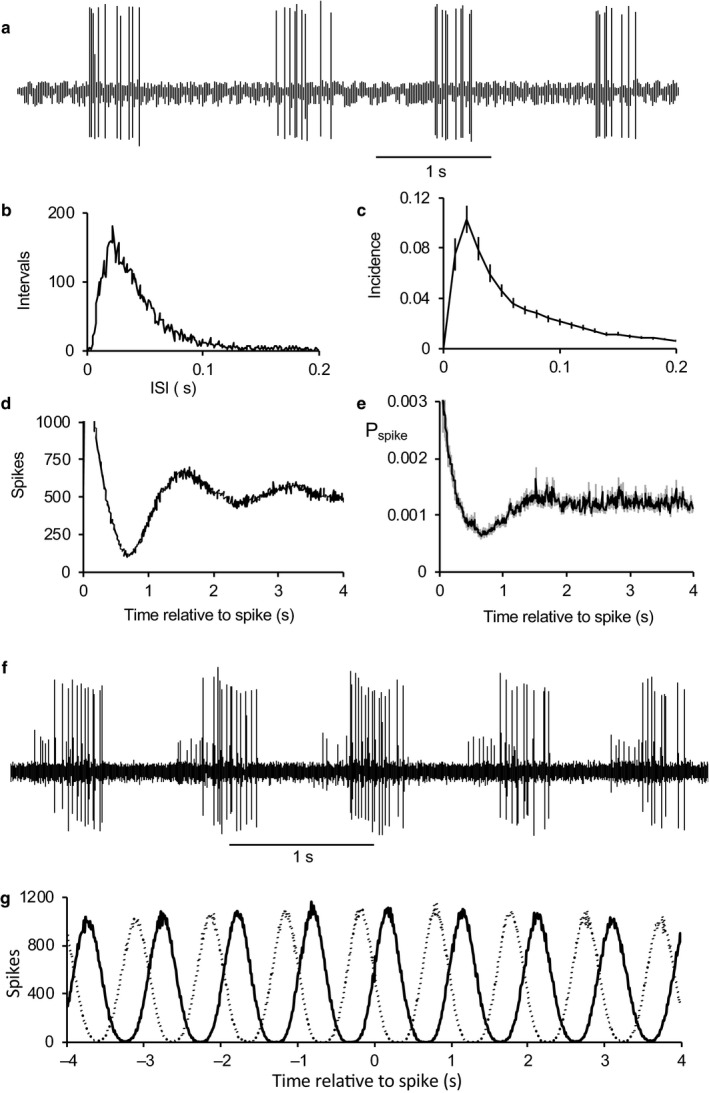
Type 2 neurons. (a) The rhythmic discharge cell of a typical type 2 neuron. The cell fired in bursts of 2–20 spikes with a period of 1.5 s. (b) ISI distribution of the neuron in A. (c) Consensus ISI distribution for 45 type 2 cells (means ± SE). (d) Autocorrelation histogram for the neuron in A,B. (e) Average normalized autocorrelations for 45 type 2 cells (means ± SE). (f) Pair of type 2 neurons, both displaying bursts with a period of about 1 s. Bursts in the smaller cell begin about 200 ms before bursts in the larger cell. (g) Cross‐correlograms (in 10 ms bins) showing activity in each of the neurons in F relative to spikes in the other, constructed over 300 s of spontaneous activity. Both neurons discharged in bursts in a rhythm with a period of about 1 s, but out of phase by about 200 ms. Bursts in cell 1 (smaller spikes in F) preceded bursts in cell 2 (larger spikes in F). The dotted line shows timing of spikes in cell 1 relative to spikes in cell 2 and the solid line shows spikes in cell 2 relative to cell A

A second subpopulation, 45 *type 2 rhythmic cells* (Figure 3) fired at 3.3 ± 0.6 spikes/s (range 0.1–21.6 spikes/s) and showed cyclic activity with a mean period approximately twice that of type 1 cells. Again, activity mainly comprised bursts of 2–10 spikes separated by ISIs of <25 ms, with sparse spiking between bursts (Figure 3a). In the typical type 2 cell shown in Figure 3a, we analyzed 911 bursts from 1352s of spontaneous activity, defining bursts by the first ISI < 300ms after an ISI of >500 ms. Bursts contained 2–25 spikes (mean (*SD*) 8(5.1) spikes; burst length 451 (287) ms; period 1.47 (0.46) s; Figure 3b). There was no frequency adaptation within bursts: the mean (*SD*) interval between the first two spikes in bursts was 49 (48) ms (*n* = 911) and that between the 15th and 16th spikes was 47 (44) ms (*n* = 96). The ISI distribution was unimodal with a mode of 23ms (Figure 3c), and the autocorrelation (Figure 3d) showed peaks at intervals of 1.2 s. The normalized average autocorrelation for all type 2 cells (Figure 3e), constructed as for type 1 cells, showed no repeated peaks as the periods varied too much between cells. Like type 1 cells, all type 2 cells had ISI distributions that were unimodal in the range 0–300 ms, with a mean mode of 17.2 ± 2.2 ms (median 13.5 ms; Figure 3f).

A third population of 35 *arrhythmic cells* fired at 6.0 ± 1.4 spikes/s (range 0.1–32.4) with no apparent oscillatory activity. The ISI distributions again were unimodal in the range of 0–300 ms, with a mean mode of 17.5 ± 2.2 ms, but activity in these cells was irregular, with no clear bursting pattern. This group includes slow firing cells where a rhythm may not have been apparent because of the low discharge rate.

### 3.2 Isoperiodic bursting

The discharge rate of many AON cells displayed intermittent long bursts superimposed on a sustained background activity. To quantify this, we measured the index of dispersion (IoD) of firing rates in 10 s bins over 300 s of spontaneous activity for each cell. For a cell firing randomly at a constant mean rate, the IoD should equal one regardless of firing rate: of the 248 cells, only 82 had an IoD below 2, while for 80 the IoD exceeded 7.

Some cells displayed repeated, long bursts from which we could measure burst and interburst durations. We analyzed 20 such cells (19 type 1 cells and 1 type 2 cell) with a mean IoD of 29.5 ± 4.9). The mean intraburst firing rate was 7.7 ± 1.0 spikes/s (median 6.7, range 1.3–19.8) for bursts of 111 ± 11 s (median 92 s, range 50–220 s). The interburst firing rates were 1.6 ± 0.4 spikes/s (median 0.9, range 0.2–7.5) for intervals of 110 ± 22 s (median 93 s, range 43–218 s). These are close to the burst and interburst durations of mitral cells in the MOB (bursts, 122 ± 10 s; interburst intervals, 129 ± 11 s), but intraburst firing rates were lower than the mean of 14.3 spikes/s in mitral cells (Leng et al., 2014).

### 3.3 Responses to social odor

**Figure 4:**
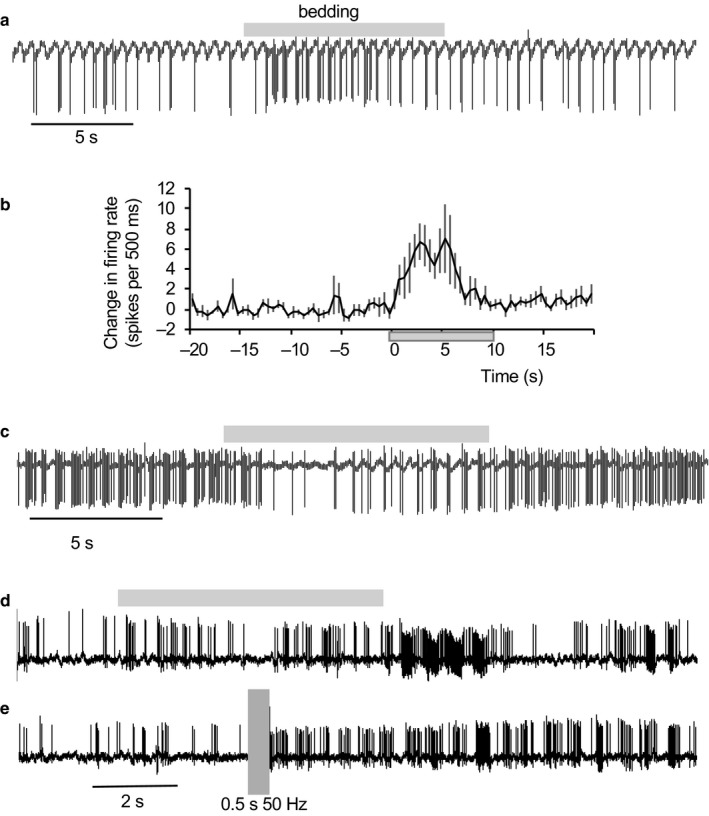
Responses of AON cells to bedding odor. (a) Example of the response of a type 1 cell to bedding odor (grey bar). (b) Averaged responses (±SE) of 10 type 1 cells that were excited by bedding odor. (c) example of an inhibitory response (one of two cells inhibited). (d) Response of a type 2 cell that was excited after the end of odor application. (e) Response of the same cell to LOT stimulation – the cell was inhibited during stimulation but showed excitation after stimulation

We applied the smell of bedding from conspecific male rats to the anesthetized male rats. Of 179 AON cells tested, 14 cells (all type 1) responded strongly and repeatedly to this but not to similar application of clean air (Figure 4); nine cells were activated (Figure 4a,b) and four were inhibited (Figure 4c). In the nine excited cells (Figure 4b), the mean firing rate increased from 3.9 ± 1.2 spikes.s^−^^1^ (in the10 s before the application) to 11.2 ± 2.6 spikes.s^−^^1^ (for 10 s during bedding application) and went back to 5.5 ± 1.8 spikes.s^−^^1^ (for 10 s after application). One cell unaffected during odor application was strongly activated after the end of odor application (Figure 4d), and two inhibited cells showed a strong excitatory rebound after the end of odor application (Figure 4e). Similar complex responses to either the presentation of an odor or to its removal were seen in other cells in response to odors other than bedding (not shown).

Seven cells activated by bedding odor responded to one or more of the other odors tested (five cells were tested with valderaldehyde of which four responded, four cells were tested with hexanol of which two responded strongly and one weakly).

### 3.4 Responses to electrical stimulation of the LOT

**Figure 5:**
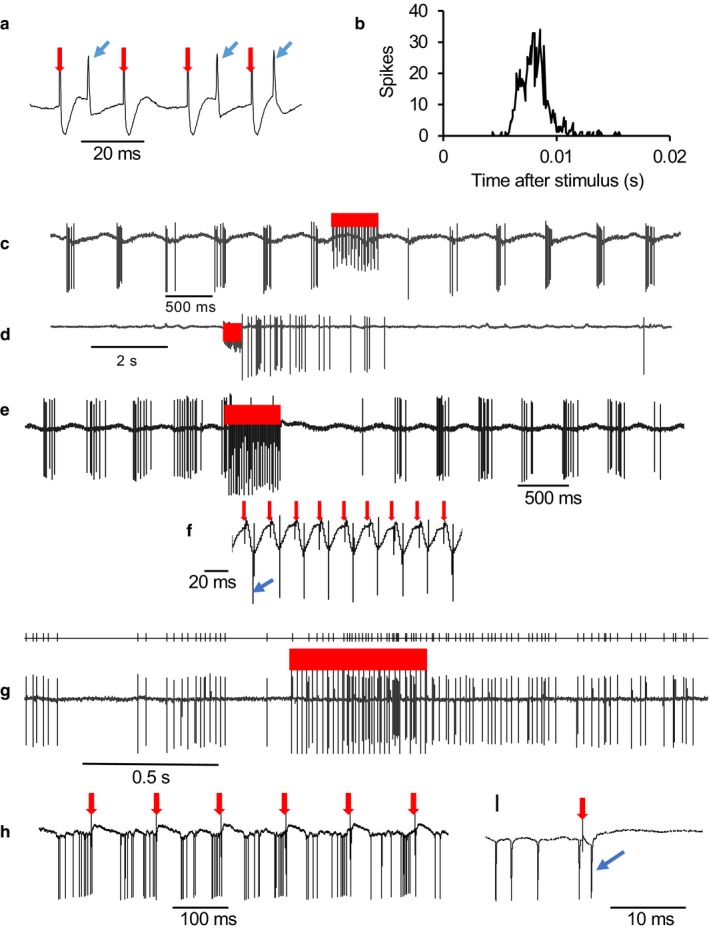
Effects of LOT stimulation on type 1 neurons. (a) Extract of voltage trace showing spike responses (blue arrows) during a train of stimuli (red arrows) at 50 Hz. (b) Post‐stimulus time histogram of the response of a type 1 neuron activated by stimulation of the LOT with a latency of about 7 ms. (c) A neuron inhibited by LOT stimulation (red bar) at 50 Hz. (d) A neuron activated after the end of LOT stimulation (red bar) (e) A neuron excited during LOT stimulation (red bar) but inhibited after. (f) Expanded voltage trace from E showing that each stimulus pulse (red arrows) is followed by a spike (the first indicated by the blue arrow). (g) A neuron progressively excited during LOT stimulation (red bar) with prolonged post‐stimulus activation. The top trace shows detected spikes. (h) This neuron was synaptically activated by LOT stimulation (red arrows) but each activation was followed by inhibition. (i) Expanded voltage trace from H showing spike (blue arrow) following stimulus (red arrow)

Of 26 type 1 cells, 10 responded to 1 Hz stimulation of the LOT with clear orthodromic excitation at latencies between 7 and 20 ms (Figure 5a,b). The other 16 were inhibited, with apparently similar latencies, although determining latency accurately in inhibited cells was generally imprecise.

In response to brief high‐frequency stimulation, 10 type 1 cells tested displayed virtually complete inhibition during stimulation (Figure 5c). In six cells, activity swiftly returned to normal after the end of stimulation, and in four there was continued inhibition for several seconds (Figure 5e). In another 12 cells, stimulation evoked a strong excitatory response. In six cases, this arose as a post‐stimulation excitation after inhibition throughout the stimulation (Figure 5d), in two cases, activation throughout stimulation was followed by a strong inhibition (Figure 5e,f,h,i). In two cases, activation began during the stimulation after initial inhibition, and outlasted the stimulation by several seconds (Figure 5g), and in two cases there was persistent activation in neurons excited throughout stimulation.

Thus, all type 1 neurons tested responded strongly to LOT stimulation, in most cases with a short latency, but had mixed inhibitory and excitatory effects, including effects that outlasted stimulation by several seconds.

**Figure 6:**
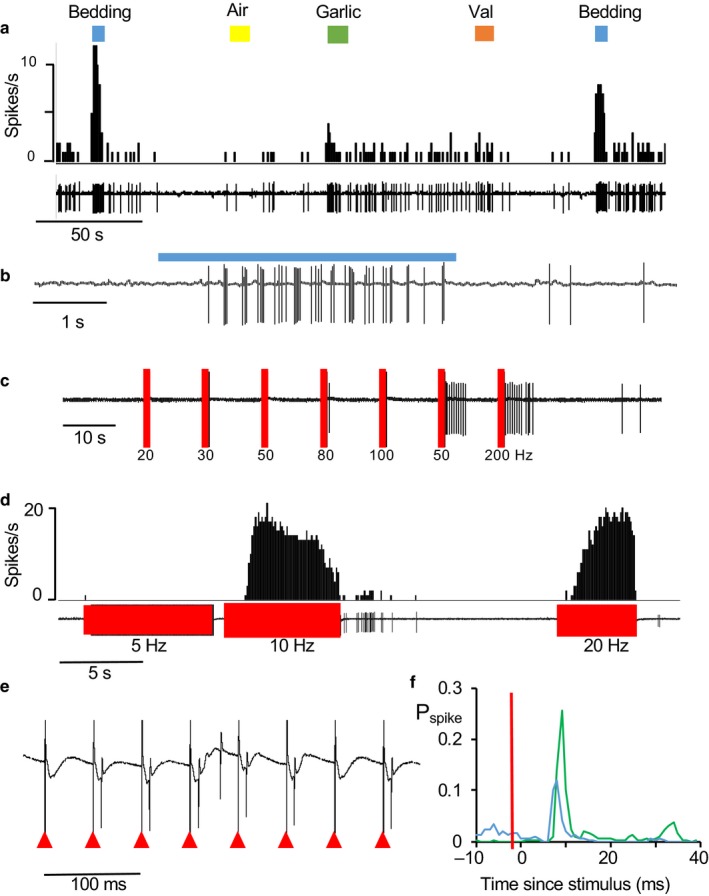
Response of a type 1 cell to odors and stimulation of the LOT. (a) This neuron was repeatably activated by bedding odor (blue bars), unaffected by air (yellow bar) or valderaldehyde (val, orange bar), and weakly activated by garlic (green bar). (b) Expansion of the response to bedding showing clear rhythmic discharge during odor application. (c) Response to trains of stimulation at the frequencies shown. During tests, the cell fell silent and was unresponsive to brief stimulation at 20, 30, and 50 Hz but was activated after stimulation at 150 and 200 Hz. (d) Responses to continued lower frequency stimulation. Stimulation at 10 and 20 Hz produced excitation after a delay. (e) Voltage record of the response during activation by 20 Hz stimulation – the stimulus artefacts are marked by the red arrowheads. (f) Peri‐stimulus histograms of responses to stimulation at 10 Hz (green) and 20 Hz (blue, expressed as the probability of a spike occurring in 1 ms bins (P_spike_). The stimulus time is marked by the red bar

Figure 6 compares the response of one type 1 cell to bedding with its response to LOT stimulation. This cell was repeatably and strongly activated by bedding odor, weakly activated by garlic, and unresponsive to air or vanilla extract (Figure 6a). The response to bedding comprised intensified cyclic bursting (Figure 6b). The same cell was tested for its response to 500 ms of LOT stimulation at between 20 and 200 Hz; it responded to stimulation at 150 and 200 Hz with a strong after‐discharge, but showed no response to lower frequencies (Figure 6c). We then tested it in response to prolonged stimulation at 5, 10, and 20 Hz (Figure 6d). Stimulation at 10 or 20 Hz was initially ineffective, but progressively revealed a short latency excitation (Figure 6e,f). This is consistent with a progressive enhancement of synaptic excitation, or with the superposition of constant excitation and a progressively waning synaptic inhibition.

**Figure 7:**
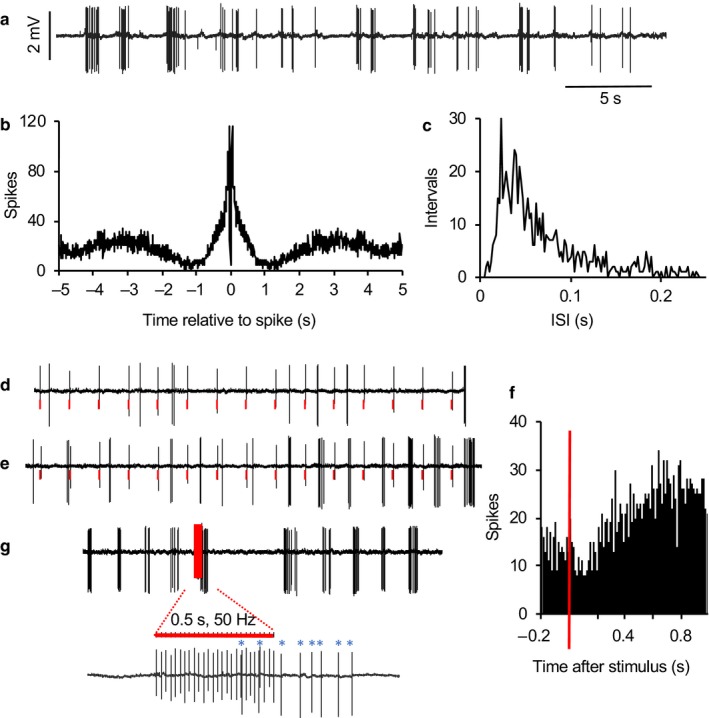
Response of a type 2 cell to stimulation of the LOT. (a) Extract of spontaneous activity of a type 2 neuron showing intermittent bursts with a period of about 2 s. (b) Autocorrelation histogram, typical of type 2 cells. (c) ISI distribution shows the typical unimodal, skewed distribution. (d) During 1 Hz stimulation of the LOT (artefacts marked in red in voltage trace), the cell initially was unresponsive. (e) With continued stimulation, bursts arose entrained by the stimuli. (f) Peri‐stimulus time histogram, revealing post‐stimulus inhibition followed by prolonged excitation. (g) The response to a brief train of stimulation at 50 Hz, showing delayed activation that outlasted the simulation

All type 2 cells tested also responded to LOT stimulation, and again the responses varied considerably. The most common responses were inhibitions followed by excitation, and the effects varied over time during continued stimulation (Figure 7). Of 13 type 2 cells tested with brief high‐frequency stimulation (50 Hz, 0.5 s), six showed strong post‐stimulus excitation and seven showed strong post‐stimulus inhibition.

### 3.5 Comparison with the respiratory cycle

Thus, these initial experiments showed that most of the AON neurons that we encountered showed strong cyclic activity presumed to reflect inputs locked to respiration, but it was clear from the occasional double recordings that different neurons fired with different phase relationships. It was also clear from the studies with LOT stimulation that some AON neurons showed short latency excitation, consistent with a direct excitatory input from the LOT. However, more neurons were inhibited by LOT stimulation, and many showed complex responses to trains of stimulation. This suggests that their responses were mainly mediated indirectly, presumably involving GABA inputs from neurons within the LOT that were themselves activated by LOT stimulation.

In subsequent experiments, we therefore focused on recording additional type 1 cells, characterized the relationship of their cyclic activity to the respiratory rhythm, and compared this with their responsiveness to LOT stimulation.

**Figure 8:**
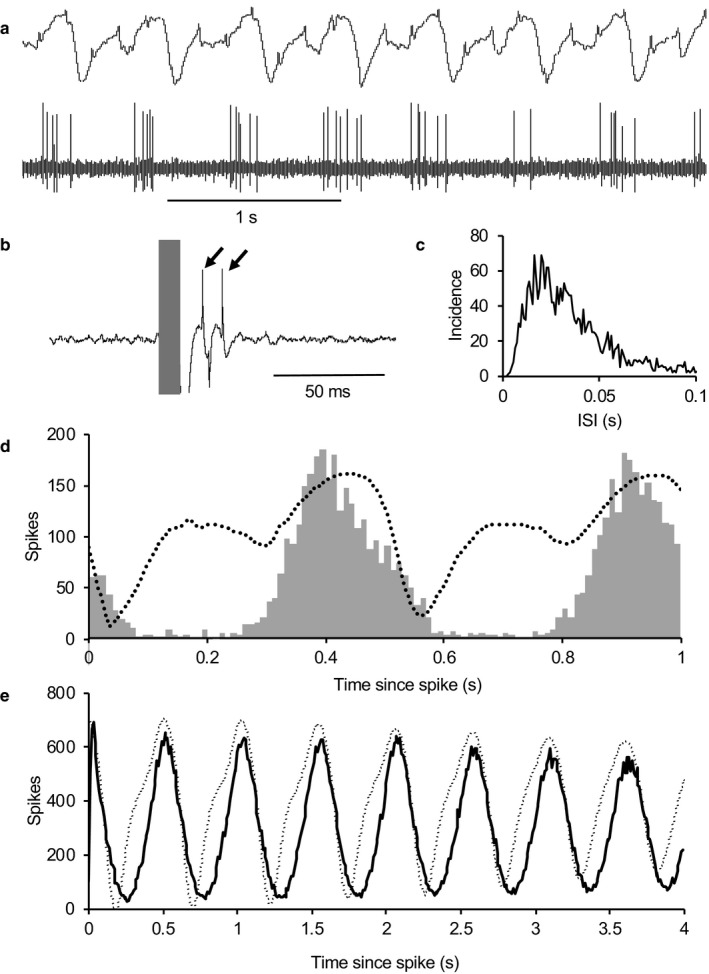
Type 1 neurons that are activated during inspiration are activated by LOT stimulation. (a) Extract of recording of respiratory activity in conjunction with recording the spontaneous activity of a type 1 neuron. (b) Identification of this neuron as activated by LOT stimulation. The shaded bar covers the stimulus artefact accompanying a stimulus pulse applied to the LOT; the pulse is followed by two spikes from this neuron (arrowed) and a smaller spike (with a negative going waveform) from a second, almost silent neuron. (c) ISI distribution from this neuron, typical of type 1 neurons. (d) Spike activity relative to respiratory cycle. During 300 s of spontaneous activity, the time of the peak inspiration in each respiratory cycle was marked, and the dotted line shows the average cycle produced from these triggers. The histogram shows the spike activity in 10 ms bins triggered in the same way. (e) Respiration relative to spike activity. The solid line shows the conventional autocorrelation histogram for this cell – this is a spike‐triggered average of spiking activity. The dotted line shows the directly corresponding spike‐triggered average of respiratory activity. Both (d) and (e) show, in slightly different ways, that the bursts in this cell were consistently initiated during inspiration

**Figure 9:**
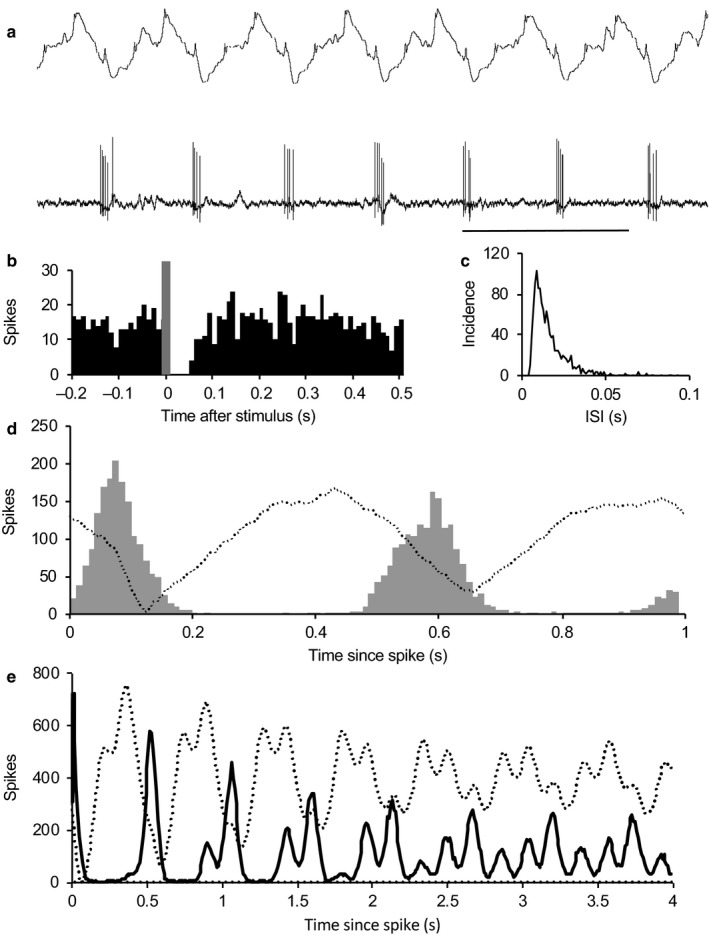
Type 1 neurons that are activated during expiration are inhibited by LOT stimulation. (a) Extract of recording of respiratory activity in conjunction with recording the spontaneous activity of a type 1 neuron. (b) Identification of this neuron as inhibited by LOT stimulation. Peri‐stimulus time histogram of spike activity (in 10 ms bins) of 300 s of activity during 1 Hz LOT stimulation. The shaded bar covers the stimulus artefact accompanying stimulation; the pulse is followed by about 50 ms of inhibition. (c) ISI distribution from this neuron, typical of type 1 neurons. (d) Spike activity relative to respiratory cycle. During 300 s of spontaneous activity, the time of the peak inspiration in each respiratory cycle was marked, and the dotted line shows the average cycle produced from these triggers (increased signal corresponds to inspiration). The histogram shows the spike activity in 10 ms bins triggered in the same way. (e) Respiration relative to spike activity. The solid line shows the conventional autocorrelation histogram for this cell – this is a spike‐triggered average of spiking activity. The dotted line shows the directly corresponding spike‐triggered average of respiratory activity. Both (d) and (e) show, in slightly different ways, that the bursts in this cell were consistently initiated during expiration

We recorded from a further 28 type 1 cells in 17 rats. For each of these, we constructed autocorrelation histograms, ISI distributions, and we used two ways to assess the relationship of spiking activity to respiratory rhythm. Nine cells showed cyclic activity beginning in the inspiratory phase of respiration, and 19 cells showed cyclic activity beginning in the expiratory phase. We could classify 20 of these neurons by their short‐latency responses to LOT stimulation; five as being excited by LOT stimulation and 15 as being inhibited. All of the five excited neurons were inspiration neurons (Figure 8); 13 of the 15 inhibited neurons were expiration neurons (Figure 9).

## 4 DISCUSSION

Processed odor information is transmitted from the MOB via the LOT to the AON (Lledo et al., 2005; Menini et al., 2004; Shipley & Ennis, 1996). The AON distributes the information to the contralateral olfactory bulb and piriform cortex and engages in reciprocal interactions with the ipsilateral bulb and cortex (Kikuta et al., 2010; Miyamichi et al., 2011; Mori, Satou, & Takagi, 1979; Sosulski et al., 2011).

From the present results, we can draw some inferences about how the AON processes the synaptic input from the MOB. In similar experimental conditions to those used here, the mitral cells and tufted cells of the olfactory bulb display rhythmic spiking activity locked to the phase of respiration. This input to the AON is purely excitatory, as mitral cells and tufted cells are all glutamatergic. Many AON cells are GABAergic, with intrinsic projections within the AON (Kay & Brunjes, 2014). Accordingly, cells in the AON are expected to be excited by LOT stimulation if they receive a direct input from the MOB (primary AON neurons), but to be inhibited if they receive inhibitory inputs from primary recipients. Of course, it is to be expected that many AON neurons will receive both direct excitatory inputs and indirect inhibitory inputs.

The first conspicuous feature of these results is that a high proportion of AON neurons display spontaneous cyclic activity tightly locked to the respiratory rhythm. This locking is apparent in the clarity of cycles displayed by the autocorrelation histograms – indeed, the cyclicity of AON neurons is more marked than that of mitral cells recorded in similar experimental conditions.

Mitral cells in the anesthetized rat alternate between long periods of activity and silence. During active phases, their mean discharge rate is 11 spikes/s, and, in cells in which a respiratory rhythm is apparent, this typically oscillates between 5 and 20 spikes/s over the respiratory cycle. AON cells are much quieter but display bursts of spikes locked to the respiratory cycle. The bursts generally comprise just a few spikes, but as these bursts are separated by long silent intervals, the periodic activity is much more marked than that of mitral cells, which are generally active throughout the respiratory rhythm. The rhythms of type 1 cells were thus more clearly defined than those of mitral cells (Leng et al., 2014) because of the sparsity of spikes between bursts. In effect, the respiratory rhythms of AON cells are more marked because the AON operates as a high‐pass filter of the MOB input.

As the respiratory rhythm must reach the AON via the output cells of the olfactory bulb, we conclude that the spontaneous cyclic activity of these AON cells is primarily driven by direct or indirect inputs from output cells of the olfactory bulb whose activity is locked to the same phase of the respiratory cycle. As stated in the introduction, there is evidence that mitral cells in the rat are generally activated late in the inspiration cycle, but not all studies have reported this; for example, Phillips *et al.* reported that mitral and tufted cells are activated at diverse phases of an artificial respiratory cycle (Phillips, Sachdev, Willhite, & Shepherd, 2012). Thus, we must consider the possibility that AON neurons that are active during inspiration receive inputs from MOB neurons that are activated during inspiration, while AON neurons active during expiration receive inputs from MOB cells that are activate during expiration. However, if so we would expect to find that AON neurons were activated by LOT stimulation regardless of how their activity was linked to respiration. Instead, we found that AON neurons that were active during inspiration were excited by LOT stimulation, whereas most of those active during expiration were inhibited by LOT stimulation.

Mitral cells typically respond to odors with either inhibition or excitation that is sustained throughout odor application, and in excited cells that display a respiratory rhythm the response involves an intensification of that rhythm (Leng et al., 2014). Mitral cells are also narrowly tuned, responding only to closely related odors. Here, we used a complex odor – the odor of male rat bedding. We chose this odor as a naturalistic odor of considerable social relevance to rats, of a type that the AON is thought to be particularly important for processing. We had previously tested this odor in our studies of mitral cells but without finding any responsive cells, possibly because we simply were not recording in responsive regions of the bulb (Leng et al., 2014): in mice, the existence of a “specialist glomerulus” narrowly tuned to a compound present in urine has been reported (Lin, Zhang, Block, & Katz, 2005). In the AON, we found quite a substantial number of cells responsive to bedding odor. The responses were generally complex, including transient “on” responses and marked “off” responses, and responsive cells were typically similarly responsive to valderaldehyde and/or hexanol – the only two “simple” odors that we tested.

A recent fMRI study in rats (Zhao et al., 2017) found no olfactory adaptation to prolonged (200 s) odor application in either the olfactory bulb or the AON, but found strong adaptation in the piriform cortex, and transient responses have recently been reported to be common for odor‐responsive neurons in the mouse piriform cortex (Tantirigama, Huang, & Bekkers, 2017). In the AON, we observed complex non‐linearities, varying between AON cells, in their response to mitral cell input, including both frequency‐dependent non‐linearities, which may amplify odor responses, and temporal non‐linearities arising presumably from slow activity‐dependent effects which in some cells produce “on” and “off” responses to odor stimuli in some cells.

We tested AON cells with brief stimulation of the LOT, which antidromically activates mitral cells and their collaterals to the AON. In some of these experiments, we stimulated at 0.5 s for 50 Hz, to mimic the activation during an inhibitory phase of the respiratory cycle as observed during odor application. Such stimulation commonly produced either a burst of activity during stimulation or an inhibition during stimulation followed by a burst after stimulation. The latter effect suggests an obvious explanation of how AON cells display rhythmic firing at different phases of the respiration cycle – as a rebound excitation following inhibition.

A feature of the AON was the many cells (the type 2 cells) showed periodic activity with a period apparently twice that of the respiratory rhythm – confirmed in examples recorded in conjunction with recordings of respiratory activity (not shown). This seems likely to occur as a result of prolonged post‐activation inhibition that occludes the excitatory input on alternate cycles. If so, the post‐activation inhibition is not plausibly accounted for by spike‐dependent after hyperpolarization intrinsic to type 2 cells, which typically discharged only a few spikes in each burst, but probably arises from aggregate inhibition by quasi‐synchronously activated interneurons.

Thus, AON neurons represent the rhythmic input from the mitral cells as periodic activation with a varied phase relationship to the original input, depending on the balance between excitation from the mitral cell input, inhibition from intrinsic neurons, and rebound excitation following inhibition, and this is reflected in the diverse phase relationships of periodic activity in adjacent mitral cells.

**Figure 10:**
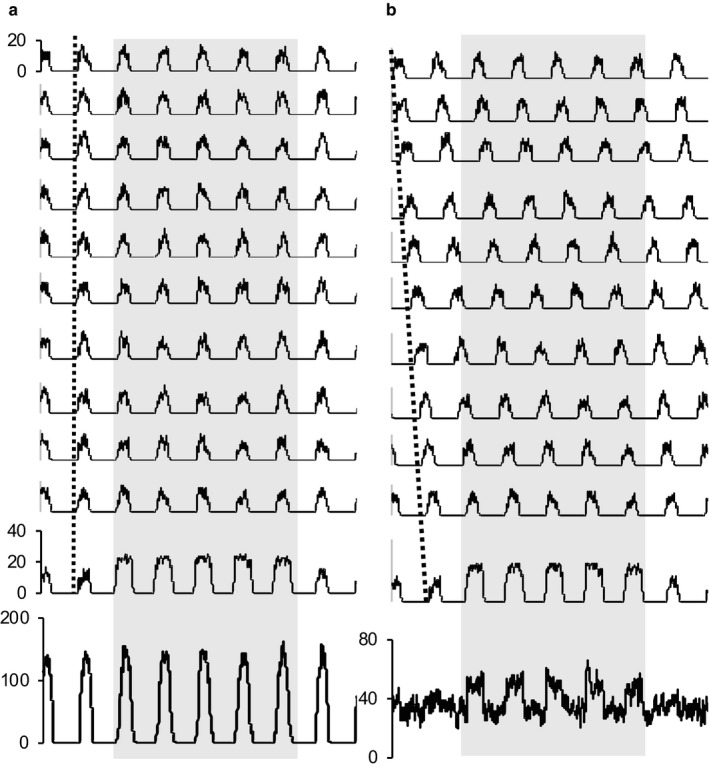
The importance of phase shifts. The mitral cells of the MOB fire spontaneously in a rhythm locked to the inspiration phase of the respiratory cycle, and each is narrowly tuned to a specific odor. In the AON, neurons receive convergent inputs from multiple mitral cells, making them more broadly tuned. However, the synchronicity of spontaneous activity rhythms presents a problem illustrated in (a). The black traces simulate the activity of 11 “cells” all firing at the same phase of the rhythm. One of these cells (the bottom trace) is activated by an odor in the period marked by the gray bar. But the aggregate activity (in gray), as seen by a second‐order neuron, the signal is virtually invisible. The signal is diluted by a factor of 1:11. In (b), the same activity of the 11 “cells” is shown, but each cell is phase shifted (dotted line). Now the signal is clearly apparent in the aggregate activity. The signal is still diluted, but by a much smaller factor of about 1:3. For simple simulation, each “cell” was generated by assuming that the probability of “spiking” was determined by a sinusoidal input

The spontaneous activity of mitral cells generally oscillates with peaks of activity in the inspiration phase of the respiratory cycle, and in the presence of an odor, MOB cells responsive to that odor discharge more intensely during inspiration. Neurons in the AON, however, receive inputs derived, directly or indirectly, from many mitral/tufted cells, only a few of which will be activated by a given odor. Considering the aggregate input, the response of the odor responsive cells will be “diluted” by the synchronous, spontaneous discharge of the non‐responsive MOB cells. Such a population is simulated in Figure 10. In this simulation, one of eleven cells is activated strongly by an “odor” to increase its activity threefold during bursts, but in the aggregate activity of the population this is imperceptible. The aggregate burst activation increases by less than 10%, within the range of random variation, and it is hard to see how any processing of this aggregate signal could reliably extract the odor signal. However, it appears that odor processing in the AON involves “phase shifting” the MOB input by variable amounts, in addition to a non‐linearity of response that yields a pattern of short bursts, linked to the respiratory cycle, with no activity between bursts. If we look at the aggregate activity of such a population (Figure 10), the response to odor stimulation of one of the 11 cells is clearly apparent as a 50% increase above a stable background rate. Not only is the signal: noise ratio protected, but the pattern of response is now readily amenable to signal extraction by neurons that response non‐linearly to this input.

In summary, the processing by AON neurons of the input from the MOB appears to include four distinct operations. First, there is convergence of inputs so that AON cells are broadly tuned. Second, processing acts as a high‐pass filter: AON neurons have a low spontaneous firing rate but because they respond non‐linearly to the MOB input, they retain a marked rhythm in the form of short bursts with a period matching the respiratory rhythm. Third, this rhythm is phase shifted in many cells, apparently by a combination of inhibition and post‐inhibitory rebound activation. This phase shifting seems likely to help maintain the signal to noise ratio of odor responses in the population output.

## CONFLICT OF INTERESTS

The authors declare that there are no conflicts of interest.

## AUTHOR CONTRIBUTIONS

C.T., T.T, Ma.L., M.L., and G.L. designed the research; C.T., T.T., and Ma.L. performed the research; T.T. and G.L. analyzed data; and G.L., T.T., C.T., and M.L. wrote the paper.
